# Assessing very advanced HIV disease in adolescent girls and young women

**DOI:** 10.4102/sajhivmed.v24i1.1501

**Published:** 2023-07-20

**Authors:** Naseem Cassim, Lindi-Marie Coetzee, Manuel P. da Silva, Deborah K. Glencross, Wendy S. Stevens

**Affiliations:** 1 Wits Diagnostic Innovation Hub (DIH), Faculty of Health Sciences, University of the Witwatersrand, Johannesburg, South Africa; 2 National Health Laboratory Service, National Priority Programme (NPP), Johannesburg, South Africa; 3 Health Science Research Office (HSRO), Faculty of Health Sciences, University of the Witwatersrand, Johannesburg, South Africa

**Keywords:** HIV, CD4, immune status, adolescent girls, young women

## Abstract

**Background:**

South Africa has the largest HIV epidemic globally, with ~7.5 million people living with HIV in 2021. Adolescent girls (AG) and young women (YW), aged 15–19 years and 20–24 years, are twice as likely to be living with HIV as their male counterparts. The national HIV prevalence for young women was 9.1% (2021), with limited data on disease severity.

**Objectives:**

This study assessed very advanced HIV disease (CD4 < 100 cells/μL) in adolescent girls and young women (AGYW) in South Africa.

**Method:**

A retrospective descriptive study analysed data collated from the National Health Laboratory Service database for 2017 to 2021 calendar years for AGYW. National and provincial specimen volumes, the percentage of tests with a CD4 < 100 cells/μL and ≥ 100 cells/μL, and the median and interquartile ranges, were calculated. Logistic regression determined the odds ratio for a CD4 < 100 cells/μL, controlling for age category.

**Results:**

Data for 1 199 010 CD4 specimens indicated a significant decrease in volumes of 34% from 287 410 (2017) to 189 533 (2021). The percentage of samples with a count < 100 cells/μL ranged from 4.9% to 5.2% for YW versus 5.6% to 6.1% for AG. Provincial data for a CD4 count < 100 cells/μL ranged between 4.5% and 8.3% in AG and 3.6% to 6.3% for YW. Logistic regression indicated a 24% higher likelihood for AG having a CD4 count < 100 cells/μL.

**Conclusion:**

The study reported a higher proportion of very advanced HIV disease for AG versus YW nationally, with provincial disparity needing further analysis.

**What this study adds:** This study provides important insights into very advanced HIV disease for adolescent girls (AG) and young women (YW). This is of extreme concern, given the reported vertical transmission rates of 3.51% in 2021. There is, however, a need to assess what other factors could result in a low CD4 count, that is, failing treatment or disengaging care.

## Introduction

Globally, an estimated 38.4 million people were living with HIV (PLWH) in 2021, of which 1.5 million people became newly infected.^1^ In addition, 650 000 deaths from AIDS-related illnesses were reported.^2^ Of PLWH, only 28.7 million (74.7%) accessed antiretroviral therapy (ART) in the same year, despite effective HIV treatment and tools to prevent, detect and treat opportunistic infections.^1,3^ In 2021, women and girls accounted for 63% of all new HIV infections in sub-Saharan Africa,^1^ with adolescent girls (AG; 15–19 years) and young women (YW; 20–24 years) accounting for 25% of new HIV infections, while only representing 10% of the population.^4^ Six out of seven new HIV infections among adolescents are among girls.^1,2^ Girls and young women aged 15 to 24 years are twice as likely to be living with HIV than young men of the same age.^1,2^

South Africa has the largest HIV epidemic globally with an estimated 7.5 million PLWH in 2021 and a national prevalence of 18.3%.^5,6^ The ART clinical guidelines for the management of HIV recommend that all PLWH are eligible to start ART, irrespective of CD4 count.^7^ As a baseline investigation, CD4 testing is required to identify eligibility for cotrimoxazole prophylaxis as well as screening for cryptococcal antigenaemia.^7^ CD4 count should be monitored for patients with virological failure at six-monthly intervals (HIV viral ≥ 1000 copies/mL).^7^ For women aged 15 to 49 years, a national prevalence of 24.5% is reported compared to 12.1% for men.^8^ Of the 210 000 new infections in 2021, the majority were in women aged 15–49 years (*n* = 130 000, 61.9%).^8^ A national prevalence of 9.1% was reported for young women (YW) and 3.0% for young men (defined as ages 15 to 24 years).^8^ Furthermore, knowledge about HIV prevention among YW in 2021 was only 46.1%.^8^ Ninety-five percent of people at risk of HIV infection should have access to an appropriate, prioritised person-centred and effective combination of prevention strategies. In addition, 90% of adolescents and young people should receive comprehensive sexuality education. HIV incidence has remained largely unchanged in the highest-burdened communities in South Africa.^9,10^ Given that adolescent girls and young women (AGYW) primarily acquire infections from older men who are often unaware of their HIV status and less likely to be on ART, the minimal decrease in HIV incidence in this sub-population in South Africa is not surprising.^10,11,12,13^ It has been reported that sexual partnering between young women and older men is a key feature of the sexual networks driving HIV transmission.^8,11^

The majority of PLWH in South Africa are aware of their status (94% based on 2021 estimates), but are diagnosed in later stages of HIV infection.^6,14^ Advanced HIV disease is defined as a CD4 cell count < 200 cells/μL and is associated with higher mortality rates due to opportunistic infections such as tuberculosis and cryptococcal meningitis.^15^ A local study in three high-burden districts in the Mpumalanga, KwaZulu-Natal and Gauteng provinces, which assessed late presentation for newly diagnosed HIV-positive individuals, reported that men, non-pregnant women and those accessing care in facilities located in townships and inner cities were more likely to present late for HIV care.^14^ HIV-positive individuals presenting late for care have an increased risk of mortality, as high as 40% of all AIDS-related deaths.^14^ Using national laboratory data, Carmona et al. reported that between 2005 and 2011, the proportion of patients entering into care with a CD4 count < 200 cells/μL declined from 46.8% to 35.6%.^13^ In contrast, from 2011 to 2016, the proportion of patients entering ART with advanced HIV disease remained relatively unchanged, ranging from 32.9% to 34.8%.^13^ The same study reported that for women seeking care, the proportion with CD4 < 200 cells/μL varied from 42.8% in 2005 to 26.5% by 2016.^13^ The study findings indicated gender disparity in late presentation between the years reported.^13^

Local studies reported that 5.4% of specimens from AGYW reported a CD4 count < 100 cells/μL for the 2019 calendar year in South Africa, defined as very advanced HIV disease.^12,13,16^ There is thus a real need for better monitoring of very advanced HIV disease, especially in a sub-population such as AGYW, already with a higher risk of infection due, in part, to risky behaviour resulting in high rates of teenage pregnancies.^17^

There is a lack of data and awareness about AGYW living with HIV. Furthermore, there is limited data for advanced HIV disease for AGYW. This study, using CD4 laboratory data for the period 2017 to 2021, aimed to describe very advanced HIV disease in AGYW.

## Methodology

### Context

Data are reported for testing performed by the National Health Laboratory Service (NHLS), with a mandate to provide laboratory services for public sector health facilities across South Africa.^18^ It has 268 laboratories across the nine provinces and serves approximately 80% of the South African population.^18^ CD4 testing is decentralised to 49 out of 268 laboratories within the NHLS and is based on an integrated tiered service delivery model.^18,19,20^

### Study design

The retrospective descriptive study design was used to analyse secondary laboratory CD4 data for the 2017 to 2021 calendar years. The sample population studied included AG (15–19 years) and YW (20–24 years) presenting for CD4 testing. Data were extracted from the Corporate Data Warehouse (CDW) of the NHLS.

### Data preparation

The following variables were provided in the extract: (1) unique patient identifier, (2) episode number, (3) age (in years), (4) ethnicity, (5) health facility, (6) province, (7) result reviewed date and (8) CD4 count value. An episode number is a unique identifier used within the laboratory information system to link specimens to a patient visit. For this work, the extract was restricted to women aged 15 to 24 years. The result reviewed date was used to extract the year and month of testing. Age in years was captured on the laboratory request form by the healthcare worker and recorded in the laboratory information system. The following age categories were assigned: (1) 15–17 years, (2) 18–19 years, (3) 20–22 years and (4) 23–24 years. Data were categorised as either AG or YW using the captured age (in years). The CD4 count was categorised as follows: (1) ≤ 10 cells/μL, (2) 11 cells/μL – 29 cells/μL, (3) 30 cells/μL – 49 cells/μL, (4) 50 cells/μL – 99 cells/μL and (5) ≥ 100 cells/μL. The CDW developed a unique patient identifier generated by a probabilistic matching algorithm that includes fuzzy logic matching.^21,22,23^ This algorithm uses the patient’s first name, last name, date of birth, gender, and hospital folder number for matching.^24^ In the data set, a patient might have had more than one test performed during the study period. The aim was to indirectly determine the extent and timeframe of, specifically, very advanced HIV disease. Therefore, data were categorised as < 100 cells/μL and ≥ 100 cells/μL. Data were prepared and analysed using SAS statistical software (Version 9.4, Cary, North Carolina, United States [US]). The logistic regression was conducted using STATA SE (STATA Corp., College Station, Texas, US).

### Statistical analysis

A decision tree was used to depict the number of specimens with a CD4 count < 100 cells/μL, with the median indicated for AG and YW. Annual test volumes for AG and YW were assessed, with the percentage change year-on-year indicated. CD4 descriptive statistics included the median and interquartile range (IQR). The data were analysed by age category, with the chi-squared test used to assess any significant associations for the CD4 category, assuming an alpha of 0.05. For AG and YW, the percentage of specimens with a CD4 count ≤ 10 cells/μL, 11 cells/μL – 29 cells/μL, 30 cells/μL – 49 cells/μL, 50 cells/μL – 99 cells/μL and ≥ 100 cells/μL for each year was calculated nationally and at the provincial level. Logistic regression was applied to determine the risk factors associated with a CD4 < 100 cells/μL (binary dependent), controlling for age category as an independent variable using STATA. The baseline comparative group was the age category 23–24 years. The odds ratio (OR), 95% confidence interval (CI) and *P*-value are reported. A *P*-value of < 0.05 indicated significant associations between the categorical variable of interest with CD4 < 100 cells/μL.

### Ethical considerations

Ethical clearance was obtained from the Human Research Ethics Committee (Medical) at the Faculty of Health Sciences, University of the Witwatersrand. The study approval number is M220163. As analyses only include secondary laboratory data, patient consent was not required. No patient identifiers were used for the study.

## Results

Of all the CD4 testing done between 2017 and 2021, 1 199 010 tests met the inclusion criteria for women aged 15 to 24 years. Following the de-duplication of data, this equates to 778 712 patients tested. Of all patients tested, 64.9% had one CD4 test during the test period, 21.5% had two tests, and 8.4% had three (i.e., 94.9% accumulatively). CD4 and patient age reported a skewness of 0.78 and –0.70. Ethnicity was provided for only 101 680 (8.5%) of the specimens.

### Decision tree analysis

**FIGURE 1:**
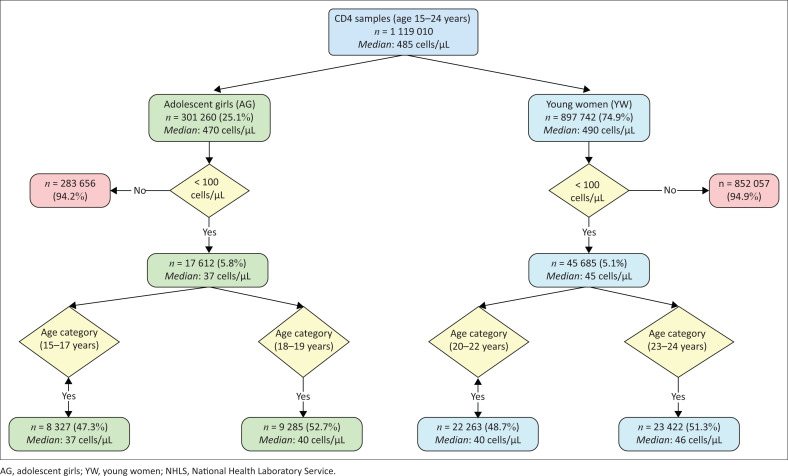
Flow chart indicating the number of specimens with a CD4 count < 100 cells/μL with the median indicated for AG and YW in South Africa for calendar years 2017 to 2021. The four age categories with median CD4 counts are indicated: (1) 15–17 years, (2) 18–19 years, (3) 20–22 years and (4) 23–24 years. Data is reported for public-sector testing by the NHLS, South Africa.

Overall, AGYW had a median CD4 count of 485 cells/μL (Figure 1). Most of the testing was done in YW (74.9%) compared to AG (25.1%). A lower median of 470 cells/μL was reported for AG compared to 490 cells/μL for YW. For AG, 5.8% of specimens tested (*n* = 301 260) for CD4 had a count < 100 cells/μL (median: 37 cells/μL) compared to 5.1% for YW (*n* = 897 742) (median: 45 cells/μL). For AGYW with a count < 100 cells/μL, a median CD4 of 34 cells/μL was reported for the 15–17 age group, 40 cells/μL for the 18–19-year-olds, 45 cells/μL for the 20–22-year-olds and 46 cells/μL for the 23–24-year-olds.

### Annual descriptive analysis

Test volumes for AGYW decreased from 287 410 to 189 533 between 2017 and 2021 (Table 1). A year-on-year percentage change of –15% was reported between 2019 and 2020. Overall, between 2017 and 2021, a percentage change of –34.1% was reported for AGYW. Between 5.1% and 5.4% of AGYW had a CD4 count of < 100 cells/μL. A median CD4 count of 475 cells/μL was reported for 2017 compared to 497 cells/μL for 2021.

**TABLE 1:** Annual analysis of CD4 testing performed for AG and YW for calendar years 2017 to 2021.

Category	2017				2018				2019				2020				2021			
	*n*	%	Median	IQR	*n*	%	Median	IQR	*n*	%	Median	IQR	*n*	%	Median	IQR	*n*	%	Median	IQR
**AG and YW**	287 410	22.6	475	303–671	254 986	20.1	486	308–692	253 368	19.9	481	305–685	213 713	16.8	493	315–697	189 533	14.9	497	315–708
% Change from preceding year	-	-	-	-	-	−11.3	-	-	-	−0.6	-	-	-	−15.7	-	-	-	−11.3	-	-
< 100 cells/μL	15 380	5.4	-	-	13 631	5.3	-	-	13 378	5.3	-	-	10 829	5.1	-	-	10 079	5.3	-	-
≥ 100 cells/μL	272 030	94.6	-	-	241 355	94.7	-	-	239 990	94.7	-	-	202 884	94.9	-	-	179 454	94.7	-	-
**AG**	69 582	24.2	462	297–659	58 909	24.5	470	299–674	64 120	25.3	466	297–666	54 640	25.6	474	305–672	50 330	26.6	480	310–682
% Change from preceding year	-	-	-	-	-	−10.0	-	-	-	2.4	-	-	-	−14.8	-	-	-	−7.9	-	-
< 100 cells/μL	4243	6.1	-	-	3687	5.9	-	-	3783	5.9	-	-	3045	5.6	-	-	2854	5.7	-	-
≥ 100 cells/μL	65 339	93.9	-	-	58 909	94.1	-	-	60 337	94.1	-	-	51 595	94.4	-	-	47 476	94.3	-	-
**YW**	217 828	75.8	479	306–675	192 390	75.5	491	311–698	189 248	74.7	486	308–692	159 073	74.4	500	319–705	139 203	73.4	503	317–717
% Change from preceding year	-	-	-	-	-	−11.7	-	-	-	−1.6	-	-	-	−15.9	-	-	-	−12.5	-	-
< 100 cells/μL	11 137	5.1	-	-	9944	5.2	-	-	9595	5.1	-	-	7784	4.9	-	-	7225	5.2	-	-
≥ 100 cells/μL	206 691	94.9	-	-	182 446	94.8	-	-	179 653	94.9	-	-	151 289	95.1	-	-	131 978	94.8	-	-

Note: AG was defined as ages 15–19 years compared to 20–24 years for YW. CD4 test volumes are reported for categories < 100 cells/μL and ≥ 100 cells/μL. Descriptive statistics (median and interquartile range) are reported for each year. The year-on-year percentage change in test volumes for AG and YW is also reported. Data are reported for public-sector testing by the NHLS, South Africa.

AG, adolescent girls; YW, young women; NHLS, National Health Laboratory Service.

For AG, testing decreased from 69 582 in 2017 to 50 330 by 2021. A year-on-year annual percentage change of between 2.4% (2019) and –14.8% (2020) was reported. Overall, AG reported a decrease of –27.7% between 2017 and 2021. The percentage of specimens with a CD4 count < 100 cells/μL ranged from 5.6% (2020) to 6.1% (2017). The median CD4 ranged between 462 cells/μL in 2017 and 480 cells/μL by 2021.

For a CD4 < 100 cells/μL, test volumes for YW decreased from 11 137 in 2017 to 7225 by 2021 (–36.1% overall reduction). Between 4.9% (2020) and 5.2% (2018) reported a count of < 100 cells/μL. The median CD4 increased from 479 cells/μL in 2017 to 503 cells/μL by 2021.

### Age category descriptive analysis

For AGYW, there were 10.7% tests performed for the 15–17 years age category, 14.4% for the 18–19-year-olds, 20–22 and 23–24 years age categories, 38.2% for the 20–22-year-olds and 36.7% for the 23–24-year-olds during the 2017 to 2021 period (Table 2). For the age categories listed, 6.5%, 5.4%, 4.9% and 5.3% of specimens reported CD4 counts < 100 cells/μL, respectively. The median CD4 ranged from 469 cells/μL (18–19 years) to 494 cells/μL (23–34 years). A *P*-value of < 0.05 (chi-squared test) was reported for the association between the CD4 and age categories.

**TABLE 2:** The CD4 test volumes for AG and YW are reported for each age category.

Category	Test volumes		< 100 cells/μL		≥ 100 cells/μL		Median	IQR	*P*
	*n*	%	*n*	%	*n*	%			
**Age category**									≤ 0.001
15–17 years	128 479	10.7	8327	6.5	120 152	93.5	471	297–673	
18–19 years	172 789	14.4	9285	5.4	163 504	94.6	469	303–668	
20–22 years	457 792	38.2	22 263	4.9	435 529	95.1	487	313–689	
23–24 years	439 950	36.7	23 422	5.3	416 528	94.7	494	310–701	

Note: The data were also categorised as < 100 cells/μL and ≥ 100 cells/μL. The median CD4 and IQR are also reported. The chi-squared test was used to evaluate the association between age and the CD4 category. Data are reported for public-sector testing between calendar years 2017 to 2021, performed by the NHLS, South Africa.

AG, adolescent girls; YW, young women; NHLS, National Health Laboratory Service; IQR, interquartile range.

### National analysis of CD4 categories by year

For the period 2017 to 2021, the percentage of AG specimens with a CD4 category of < 10 cells/μL was 1.2%, 1.3% for 11 cells/μL – 29 cells/μL, 1.0% for 30 cells/μL – 49 cells/μL and 2.3% for 50 cells/μL – 99 cells/μL (data not shown). In comparison, for YW, 0.8%, 1.0%, 0.9%, and 2.4% were reported for the CD4 categories as indicated.

**FIGURE 2:**
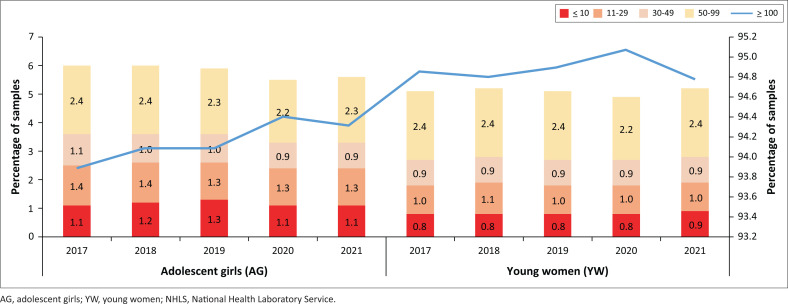
National analysis of the percentage of CD4 specimens with a count ≤ 10 cells/μL, 11 cells/μL – 29 cells/μL, 30 cells/μL – 49 cells/μL, 50 cells/μL – 99 cells/μL and ≥ 100 cells/μL by calendar year for AG and YW between 2017 and 2021. AG was defined as ages 15–19 years compared to 20–24 for YW. The ≥ 100 cells/μL category is reported on the secondary *y*-axis. Data are reported for public-sector testing by the NHLS, South Africa.

For AG, a range of 93.9% (2017) to 94.4% (2020) had a CD4 count ≥ 100 cells/μL (Figure 2). In comparison, for YW, between 94.8% (2018) and 95.1% (2020) reported a count ≥ 100 cells/μL.

For the ≤ 10 cells/μL category, a range of 1.1% – 1.3% was reported for AG compared to 0.8% – 0.9% for YW. For AG, a range of 1.3% – 1.4% was reported for ranges of 11 cells/μL – 29 cells/μL, 0.9% – 1.1% for 30 cells/μL – 49 cells/μL, and 2.2% – 2.4% for 50 cells/μL – 99 cells/μL. Ranges of 1.0% – 1.1%, 0.9% – 0.9% and 2.2% – 2.4% were reported for YW in the same CD4-range categories (Figure 2).

### Provincial analysis of very advanced HIV disease (CD4 < 100 cells/μL)

**FIGURE 3:**
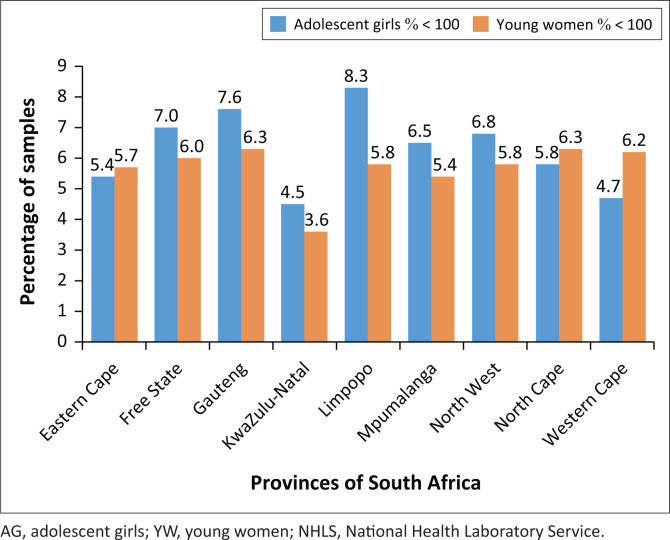
Provincial analysis of the percentage of CD4 specimens with a count ≤ 100 cells/μL for AG and YW for the period from 2017 to 2021. Data are reported for public-sector testing by the NHLS, South Africa.

The provincial analysis revealed that for AG, between 4.5% (KwaZulu-Natal) and 8.3% (Limpopo) reported a count < 100 cells/μL. Between 3.6% (KwaZulu-Natal) and 6.3% (Northern Cape) of YW had a count < 100 cells/μL (Figure 3). Nationally, 5.3% of AGYW reported a CD4 count < 100 cells/μL. Two provinces, KwaZulu-Natal (4.5%) and the Western Cape (4.7%), reported ≤ 5.3% of AG with a count < 100 cells/μL. The percentage of specimens with a count < 100 cells/μL below the national value of 5.3% for YW was only reported for KwaZulu-Natal (3.6%).

### Association between a CD4 count < 100 cells/μL and age category

For the 15–17 years age category, an odds ratio of 1.24 (CI: 1.21–1.27) was reported, 1.00 (CI: 0.98–1.03) for 18–19-year-olds and 1.90 (CI: 0.89–0.92) for 20–22-year-olds (Table 3). These results indicate that the 15–17 years age group is 24% more likely to have a CD4 < 100 cells/μL (significance, *P* ≤ 0.0001). In comparison, the 20–22 years age group is 10% less likely to have a CD4 count < 100 cells/μL (significance, *P* ≤ 0.0001). No significant difference was noted for the 18–19 years age group when compared to the 23–24 years age group (*P* = 0.693).

**TABLE 3:** Logistic regression to assess the association between specimens with a CD4 < 100 cells/μL (dependent binary variable) and age category (independent variables) between 2017 and 2021.

Characteristic	OR	95% CI	*P*
**Age category**			
15–17 years	1.24	1.21–1.27	< 0.0001
18–19 years	1.00	0.98–1.03	0.693
20–22 years	0.90	0.89–0.92	< 0.0001
23–24 years	1.00	-	-

Note: Data are reported for public-sector testing for AG and YW by the NHLS, South Africa.

For the logistic regression, the OR, 95% CI and *P*-value were reported. An alpha of 0.05 was assumed.

OR, odds ratio; 95% CI, 95% confidence interval; AG, adolescent girls; YW, young women; NHLS, National Health Laboratory Service.

## Discussion

The proportion of specimens with very advanced HIV disease was highest for those aged 15 to 17 years, despite reporting a higher median CD4. Overall, for AGYW, levels of very advanced HIV disease remained above 5% between 2017 and 2021, despite significant changes in test volumes. The decrease in test volumes may be due in part to guideline changes,^7^ and the impact of coronavirus disease 2019 (COVID-19).^10,25^ Any proportion of AGYW presenting late for care is of extreme concern where vertical transmission rates are as low as 3.51%.^8^ At the national level, the proportion of specimens (the vast majority adults of 18 years and older) representing very advanced HIV disease was 11.5% (2018), 11.4% (2019), 10.8% (2020) and 10.8% (2021) (data not shown).^18,26,27^ However, lower levels of very advanced HIV in AGYW compared to national data suggest that enhanced focus on this group may lead to early diagnosis and treatment.^18,26,27,28^

This study reveals high rates of very advanced HIV disease, suggesting HIV seroconversion in much younger girls, especially worrying bearing in mind the trajectory of HIV disease progression.^29,30^ It has been reported that AGYW are at the epicentre of the HIV epidemic in southern Africa, contributing a disproportionate ~30% of new infections and seroconverting between 5 and 7 years earlier than their male peers.^31^ Evidence-based specific healthcare interventions for AGYW, such as the determined, resilient, empowered, AIDS-free, mentored and safe (DREAMS [determined, resilient, empowered, AIDS-free, mentored and safe]) initiative, aim to reduce new HIV infections by empowering AGYW to reduce their risk, strengthen families, mobilise communities for change and reduce the risk in men who are likely to be their male sex partners.^32,33^ The goals of the DREAMS initiative are to ensure that AGYW have access to prevention technologies and strategies, and the opportunity to complete high school and graduate HIV-negative and without pregnancies.^32,34^ This approach should hopefully address AGYW at the highest risk of HIV acquisition through political commitment, leadership, financial and human resource investments, advocacy efforts, and a focus on the highest priority settings.^10^ Based on our findings, these interventions should be focussed on those aged 15–17 years who were more likely to have very advanced HIV disease. However, prevention intervention should be targeted at earlier age groups with very advanced HIV disease.

A myriad of factors are associated with HIV infection vulnerability among AGYW.^32^ Recent systematic reviews cite a history of sexually transmitted infections, alcohol use, multiple partners, early marriage, being out of school, inconsistent condom use and engaging in transactional sex^32^ where AGYW primarily acquire infections from older HIV-positive men (20s and early 30s).^10^ It has been reported by Karim et al. that AGYW have the least power in society, and bear an enormous burden of both intimate partner violence (IPV) and HIV.^35^ A key intervention to reduce gender-based violence (GBV) is primary and secondary education of AGYW.^35,36^ This may reduce rates of HIV infection, delay childbearing, lower infant and maternal mortality rates, and improve other development outcomes.^35,37^ Bearing the disparity in HIV prevalence between AGYW (9.1%) and young men (3.0%) in mind, it appears that GBV and IPV are important drivers of HIV transmission for AGYW and unintended pregnancies.^38^ However, there is an interplay of psychosocial gender-power disparities and socioeconomic and other social anomalies which lead to vulnerability in women in Africa.^10^

The provincial data revealed similar outcomes to national data. For AG, higher levels of very advanced HIV disease in the Free State, Gauteng and Limpopo provinces (above 7%) is a concern. Similarly, over 6% of testing for YW reported CD4 counts < 100 cells/μL in the Free State, Gauteng, Northern Cape and Western Cape provinces. These findings indicate provincial disparities in levels of very advanced HIV disease in YW. This is similar to findings reported by two separate local studies assessing late presentation.^13,28^ A local study reported that the coverage of prevention of mother-to-child transmission ART (dual prophylaxis or nevirapine at birth) ranged from 63.9 (Eastern Cape) to 87.4% in KwaZulu-Natal.^39^ Our data showed a correlation between very advanced disease in AG in Limpopo and Mpumalanga provinces with mother-to-child-transmission data.^39^ For YW, a correlation was found for Eastern Cape, Limpopo and Mpumalanga provinces.^39^

To understand why AGYW are presenting late for HIV care, further analysis is required at the health district and sub-district levels.^40^ This would include integrating clinical and laboratory aggregate findings to clearly understand why and where late presentation for AGYW persists. Unfortunately, without matching clinical data it is not possible to assess access to care and longitudinal follow-up. The integration of clinical and laboratory data would be critical to developing longitudinal cohorts and providing outcomes to laboratory data. Once a clearer understanding is obtained from the data analysis, policymakers can develop and implement focussed programmatic interventions.

### Limitations

The study used laboratory data to assess very late presentation in AGYW. Due to the absence of matching clinical data, it is not possible to determine whether CD4 testing was performed for baseline clinical/laboratory evaluation before ART.^7^ It is not possible to determine whether CD4 testing was performed for AGYW on ART.

The study findings reveal that testing specifically for AGYW decreased year-on-year since 2017, with reductions of up to 15.7% reported between years. In comparison, for national CD4 data (all ages), a percentage reduction of between 5% and 8% annually is noted (data not shown). The change in HIV guidelines, that is, universal test and treat, is one of the contributing factors to the annual decrease in CD4 test volumes. Comparative results spanning across 10 years of CD4 testing (2012 to 2022) indicate that the percentage of 15–24-year-old women’s contribution to total CD4 tests has not changed significantly (< 2%) over time (data in preparation for publication). As a response to limit the spread of severe acute respiratory syndrome coronavirus 2 infections, the implementation of social distancing and lockdown measures in 2020 led to restrictions in healthcare accessibility and may have contributed to the declines seen from 2020 but was not a contributor to prior yearly declines. Mid-year population estimated between 2017 and 2021 indicated a decrease of 6.3% reported for AG compared to 6.7% for YW (data not shown).^41^ However, study findings of year-on-year decreases in testing for AGYW do not match the reported decrease in population estimates,^41^ that is, between 2019 and 2020, a 15.7% testing reduction was reported for AGYW compared to a 0.2% reduction for mid-year population estimates.^41^ Possible contributing factors to the steady yearly decline could relate to the non-youth-friendly perception of healthcare facilities and healthcare worker biases regarding the provision of sexual health services to AGYW.^31,42^ Data are also not available to assess whether AGYW were on treatment and whether any programmatic interventions had taken place. This would require the integration of clinical and laboratory data systems not currently in place in South Africa.^43,44,45^

An important limitation is that some of the AGYW presenting with very advanced HIV disease were infected at birth (vertical transmission).^46^ National and provincial representative surveys in South Africa have reported a reduction of the vertical transmission of HIV from > 25% in the absence of vertical transmission prevention interventions to 2.6% by 2012–2013.^46^ Unfortunately, data for mothers who present for delivery without having accessed antenatal/prenatal care are not available. Although the probabilistic matching algorithm was used, we would require earlier data to conduct a first-ever CD4 analysis, which is out of the scope of this study. Work is underway to develop an extended HIV cohort algorithm that would make it possible to analyse first-ever CD4 trends. This may help to identify vertical transmission (using HIV DNA polymerase chain reaction testing), with follow-up CD4 testing when the AGYW present for pre-ART HIV care.

One of the limitations of this study is that it only reported the immunological status of AGYW presenting for CD4 testing. Correlating their immune status to their clinical staging was out of the scope of this study. Data analysis was further subject to the accuracy of patient age captured on the request form by the healthcare worker and laboratory clerk.

## Conclusion

This study demonstrates the value of utilising secondary laboratory data (stratified by age and gender) to identify potential weaknesses in the healthcare of individuals with very advanced HIV disease. This data have made it possible to describe unacceptable levels of very advanced HIV disease in sub-populations such as AGYW. The provincial disparity of very advanced HIV disease for AGYW indicates that further analysis is required with clinical data to elucidate where interventions are required. It is important to focus on interventions that ensure that AGYW have access to appropriate healthcare services, prevention technologies and strategies, and complete high school and graduate HIV-negative.^32^ The integration of clinical and laboratory data would be critical to develop longitudinal cohorts and provide outcomes to laboratory data.
